# Molecular Evidence of the Toxic Effects of Diatom Diets on Gene Expression Patterns in Copepods

**DOI:** 10.1371/journal.pone.0026850

**Published:** 2011-10-28

**Authors:** Chiara Lauritano, Marco Borra, Ylenia Carotenuto, Elio Biffali, Antonio Miralto, Gabriele Procaccini, Adrianna Ianora

**Affiliations:** Stazione Zoologica Anton Dohrn, Napoli, Italy; Institute of Marine Research, Norway

## Abstract

**Background:**

Diatoms are dominant photosynthetic organisms in the world's oceans and are considered essential in the transfer of energy through marine food chains. However, these unicellular plants at times produce secondary metabolites such as polyunsaturated aldehydes and other products deriving from the oxidation of fatty acids that are collectively termed oxylipins. These cytotoxic compounds are responsible for growth inhibition and teratogenic activity, potentially sabotaging future generations of grazers by inducing poor recruitment in marine organisms such as crustacean copepods.

**Principal Findings:**

Here we show that two days of feeding on a strong oxylipin-producing diatom (*Skeletonema marinoi*) is sufficient to inhibit a series of genes involved in aldehyde detoxification, apoptosis, cytoskeleton structure and stress response in the copepod *Calanus helgolandicus*. Of the 18 transcripts analyzed by RT-qPCR at least 50% were strongly down-regulated (aldehyde dehydrogenase 9, 8 and 6, cellular apoptosis susceptibility and inhibitor of apoptosis IAP proteins, heat shock protein 40, alpha- and beta-tubulins) compared to animals fed on a weak oxylipin-producing diet (*Chaetoceros socialis*) which showed no changes in gene expression profiles.

**Conclusions:**

Our results provide molecular evidence of the toxic effects of strong oxylipin-producing diatoms on grazers, showing that primary defense systems that should be activated to protect copepods against toxic algae can be inhibited. On the other hand other classical detoxification genes (glutathione S-transferase, superoxide dismutase, catalase, cytochrome P450) were not affected possibly due to short exposure times. Given the importance of diatom blooms in nutrient-rich aquatic environments these results offer a plausible explanation for the inefficient use of a potentially valuable food resource, the spring diatom bloom, by some copepod species.

## Introduction

Diatoms are dominant photosynthetic organisms in the world's oceans and are considered essential in the transfer of energy through marine food chains including important fisheries. However, numerous studies have shown that these unicellular plants at times produce secondary metabolites with toxic effects on reproductive processes in crustacean copepods [1], [2], [3] and cladocerans [4], echinoderm sea urchins [5] and sea stars [6], [7], polychaete worms [8], [9], and ascidians [10]. Diatom metabolites are the end-products of a lipoxygenase/hydroperoxide lyase metabolic pathway [11], [12], [13], [14], [15] initiated by damage to algal cells, as occurs through grazing by predators. Cell damage activates lipase enzymes, which liberate polyunsaturated fatty acids (PUFAs) from cell membranes that are immediately oxidized and cleaved within seconds to form polyunsaturated aldehydes (PUAs) and a plethora of other metabolites collectively termed oxylipins.

Oxylipins, and PUAs in particular, have important biological and biochemical properties including the disruption of gametogenesis, gamete functionality, fertilization, embryonic mitosis, and larval fitness and competence [7]. Although the effects of such toxins are less catastrophic than those inducing poisoning and death of predators, they are none-the-less insidious inducing abortions, birth defects and reduced larval survivorship [1], [16]. Such antiproliferative compounds may discourage herbivory by sabotaging future generations of grazers, thereby allowing diatom blooms to persist when grazing pressure would otherwise have caused them to crash. Similar wound-activated compounds are also found in terrestrial plants where they play a pivotal role in defense because of their antibacterial, wound healing and antiproliferative activity [17].

In a recent study [18] the authors showed that alpha and beta tubulin gene expression levels were significantly reduced when females of the copepod *Calanus helgolandicus* were fed on the ubiquitous diatom-blooming species *Skeletonema marinoi* (*S. marinoi*) which is known to produce high quantities of PUAs and several other oxylipins including fatty acid hydroperoxides, hydroxyl- and keto-fatty acids, and epoxyalcohols [3]. The aim of the present study was to further explore the toxic effects of diatoms on copepod females at the gene level under two different experimental conditions: when females were fed for 2 days (2d) on *S. marinoi* compared to when they received a diet of another diatom *Chaetoceros socialis* (*C. socialis*) that does not produce PUAs and synthesizes only low levels of other oxylipins [3], and was thus, in theory, “less toxic” for copepods.

**Figure 1:**
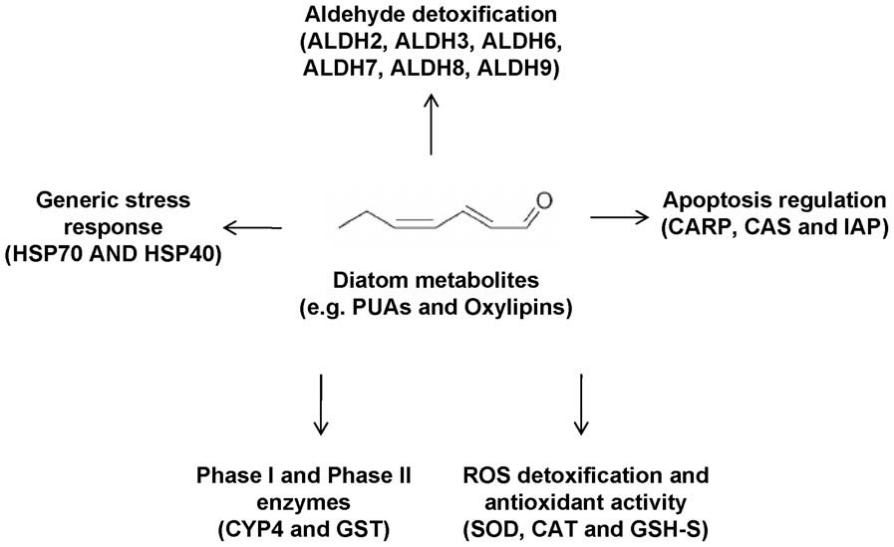
Putative systems affected by diatom metabolites in the copepod *Calanus helgolandicus.* A synopsis of the defense and detoxification systems and generic stress response (with selected genes in parenthesis) studied in *C. helgolandicus* females exposed to different diatom diets (*Skeletonema marinoi* and *Chaetoceros socialis*). Selected genes were six Aldehyde dehydrogenases (ALDH2, ALDH3, ALDH6, ALDH7, ALDH8, ALDH9), Cytochrome P450-4 (CYP4), Catalase (CAT), Superoxide Dismutase (SOD), Glutathione S-Transferase (GST), Glutathione Synthase (GSH-S), Inhibitor of Apoptosis Protein (IAP), Cell Cycle and Apoptosis Regulatory 1 Protein (CARP), Cellular Apoptosis Susceptibility Protein (CAS) and Alpha and Beta tubulins (ATUB and BTUB).

In addition to the previously investigated alpha and beta tubulins, here we analyzed the effects of these two diatom diets on the expression levels of genes which are known to have a primary role in generic stress responses, defense systems (e.g. aldehyde, free fatty acid and free radical detoxification) or apoptosis regulation in other organisms, from humans to marine organisms [19], [20], [21], [22], [23], [24], [25], [26], [27] (Figure 1). We expected an activation of enzymes and proteins involved in stress responses (e.g heat shock proteins, phase I and phase II enzymes), but, in particular, we hypothesized expression level increases of enzymes that could detoxify and/or metabolize toxic diatom PUAs.

To study the generic stress response of *C. helgolandicus* to diatom toxicity, we analyzed the heat shock protein families 40 and 70 (HSP40 and HSP70, respectively). HSPs are highly conserved proteins that are activated in response to various environmental stress factors [28], [29]. HSP70 can be involved in the tolerance of hyperthermia, ischemia/hypoxia, resistance to hydrogen peroxide, escape from drug-induced cell cycle arrest, tolerance to ultraviolet radiation and apoptosis [28] whereas HSP40 is often co-localized with HSP70 and plays a role in regulating the ATPase activity of HSP70 [30].

Since oxylipins induce LPO and an increase in free fatty acids [31], we also analyzed the microsomal cytochrome P450 family 4 monooxygenases (CYP4) that generally catalyze the ω-hydroxylation of fatty acids, arachidonic acid and derivatives such as leukotrines and prostanoids [32]. Cytochrome P450 (CYP) enzymes, especially members of the CYP1, CYP2, CYP3, and CYP4 families are generally involved in oxidative modification (known as Phase I reaction) of chemicals into more hydrophilic metabolites to enhance their elimination or inactivation [33].

Diatom oxylipins are also known to induce an increase in free radicals, such as reactive oxygen species (ROS) [3] which induce oxidative stress and damage to DNA, RNA, proteins, lipids and carbohydrates. We therefore analyzed enzymes involved in ROS detoxification, such as catalase (CAT) and superoxide dismutase (SOD) [34] and antioxidant activity, such as glutathione synthase (GSH-S) and glutathione S-transferase (GST). Glutathione is an important cell scavenger molecule which facilitates dis-activation of radical compounds. GST is involved in phase II detoxification reactions, catalyzing the nucleophilic attack of glutathione on electrophilic substrates, decreasing their reactivity with cellular macromolecules, facilitating dissolution of the complex glutathione-substrate in the aqueous cellular and extracellular media and, consequently, its elimination from the body [35].

We also analyzed aldehyde dehydrogenases (ALDHs) which constitute a superfamily of enzymes that catalyze the oxidation of endogenous and exogenous aldehydes into their corresponding carboxylic acids. These enzymes are generally involved in many processes including amino acid catabolism, neurotransmitter metabolism, xenobiotic and drug biotransformation, protection from osmotic stress and detoxification reactions [26], [36]. We selected several aldehyde dehydrogenase isoforms (ALDH2, ALDH3, ALDH6, ALDH7, ALDH8 and ALDH9) that are mainly involved in aldehyde detoxification due to lipid peroxidation (LPO) [26], [37], [38].

Previous studies have shown that PUAs and oxylipins also induce apoptosis and teratogenesis in the offspring of female copepods that have fed on diatoms for ≥5 d [16]. We therefore determined the transcription level of a protein belonging to the Inhibitor of apoptosis family (IAP), the cell cycle and apoptosis regulatory 1 protein (CARP) and the cellular apoptosis susceptibility protein (CAS). IAP levels increase in certain tumors probably contributing to resistance to apoptosis [39], CAS and CARP are also both involved in apoptosis [22], [40], [41]: CARP is a novel cell growth regulator [42] and CAS is necessary in the mitotic spindle checkpoint that ensures genomic stability during cell division [41]. In addition, we also analyzed microtubule subunits (alpha and beta tubulins), necessary for mitotic spindle formation. Microtubules (MTs) have many other cellular functions including development and maintenance of cell shape, growth, signaling, protein movement, intracellular vesicle transport and organization and positioning of membranous organelles [43], [44], [45], [46].

## Materials and Methods

### Microalgae culture

The planktonic diatoms *Skeletonema marinoi* (SMFE6; Adriatic Sea isolate FE6) and *Chaetoceros socialis* (CSFE17) were cultured as described in [47] and harvested during the stationary growth phase. Both species are part of the culture collection at the SZN. SMFE6 produces the PUAs 2-trans-4-cis-hepta-2,4-dienal as the dominant compound with smaller quantities of 2-trans-4-cis-octa-2,4-dienal and 2-trans-4-cis-octa- 2,4,7-trienal as well as a number of other products deriving from the oxidation of fatty acids including 9S-hydroxy-hexadecatrienoic acid, 11,9-hydroxy-epoxy-hexadienoic acid, 9S-hydroxy-hexatetraenoic acid, 5R- and 15S-hydroxy-eicosapentaenoic acids and 13,14S-hydroxy-epoxy-eicosatetraenoic acid, as described in [3], [47]. CSFE17 produces 9S-hydroxy-eicosapentaenoic acid, 9S-hydroperoxy-eicosapentaenoic acid and 7,8-hydroxy-epoxy-eicosatetraenoic acid but not PUAs [3].

### Copepod Feeding Experiments

*Calanus helgolandicus* specimens were collected in the North Adriatic Sea and transported to Naples where they were placed in a 500 L re-circulating copepod breeding system [48]. *C. helgolandicus* adult females were isolated under a Leica stereomicroscope, transferred to 1000 ml bottles (about 15–20 copepods/bottle) filled with 0.22 µm filtered sea water (FSW) at 20°C and fed either unialgal diets of the control flagellate *Rhodomonas baltica* (7500–8000 cells/ml) (which does not produce any oxylipins) or the test diatoms *S. marinoi* (45.000–60.000 cells/ml) and *C. socialis* (48.000–55.000 cells/ml) for two days (2 d). After 2 d, copepods were transferred to clean bottles with FSW for 24 h to eliminate any algal residues in the gut. For each diet, triplicate samples of 5 animals each were carefully transferred to 500 µl Trizol Reagent (Invitrogen), frozen directly in liquid nitrogen and stored at −80°C until RNA extraction.

### RNA extraction and cDNA synthesis

Total RNA was extracted from each copepod replicate according to Trizol manufacturer's protocol (Invitrogen). Each sample was treated with DNaseI (Invitrogen) according to the instruction manual to remove hypothetically contaminating DNA. RNA quantity and purity was assured by Nano-Drop (ND-1000 UV-Vis spectrophotometer; NanoDrop Technologies), RNA quality by gel electrophoresis. 1 µg of each RNA sample was retro-transcribed in complementary DNA (cDNA) (doublestrand DNA version of an mRNA molecule) with the iScriptTM cDNA Synthesis Kit (BIORAD) following the manufacturer's instructions, using the GeneAmp PCR System 9700 (Perkin Elmer). The reaction was carried out in 20 µl final volume with 4 µl 5× iScript reaction mix, 1 µl iScript reverse transcriptase and H_2_O. The mix was first incubated 5 min at 25°C, followed by 30 min at 42°C and finally heated at 85°C for 5 min.

### Primer design

Primers were designed considering the alignment of conserved domains in other species. Table 1 lists primers' sequences, amplicon size, correlation coefficient (R^2^) and efficiency (E). PCR conditions were optimized on a GeneAmp PCR System 9700 (Perkin Elmer). For a detailed description see [18]. Amplified PCR product sequences are deposited in GenBank under the Accession Numbers shown in Table 1.

**Table 1:** Reference Gene and Genes of Interest in the copepod *Calanus helgolandicus* RT-qPCR assays.

Acronym	Gene name	Acc. no.	Primer Forward (5′-3′)	Primer Reverse (5′-3′)	Amplicon size	E	R^2^
S20	Ribosomal protein S20	HQ270531	CGTAAGACTCCTTGTGGTGAGG	GAAGTGATCTGCTTCACGATCTC	113	89%	0.9915
ATUB	Alpha tubulin	HQ270529	ACAGCTTCTCCACCTTCTTCTC	GTTGTTGGCGGCATCCTC	167	94%	0.9997
BTUB	Beta tubulin	HQ270528	GGATTTCAGCTGACCCACTC	GTCTCATCAGTATTTTCCACCAG	205	97%	0.9862
CYP4	Cytochrome P450-4	JF825512	CTGATCACTCCAACTTTTCACTTC	CCATTGCAGTCTCACAGATTATG	169	100%	0.959
ALDH2	Aldehyde Dehydrogenase 2	JF825506	GGACAAGGCAGATGTCAACAA	ATAGGGTTTGCCATTGTCAAG	181	100%	0.998
ALDH3	Aldehyde Dehydrogenase 3	JF825507	CCTCTTGGTGTTGTCCTGATC	CCAACTCTGATGGCTTGATG	117	95%	0.997
ALDH6	Aldehyde Dehydrogenase 6	JF825508	GAGCAGTGCTGCAGCAACAC	GGAACATCCAGAGGGGGATC	164	100%	0.989
ALDH7	Aldehyde Dehydrogenase 7	JF825509	CAGGAGTATGTTGACATCTGTGAC	GAAGTTGAAGGCGGTGATG	154	100%	0.988
ALDH8	Aldehyde Dehydrogenase 8	JF825510	CTGGAGGAGTTTGCAGTGG	GCCAGCCACACCAATAGG	198	100%	0.997
ALDH9	Aldehyde Dehydrogenase 9	JF825511	GGAAAACCAATCTGGGAAGC	CAAAGGGTAGTTCCAGGCTC	183	100%	0.988
GST	Glutathione S-Transferase	JF825513	CAACCCCCAGCACACTGTG	GGATAGACACAATCACCCATCC	210	83%	0.992
GSH-S	Glutathione Synthase	JF825516	GAGAAGGCAAAGGACTATGCTC	GGCAACCTTGTGCATCAAC	180	97%	0.998
CAT	Catalase	JF825517	TGTACATGCAAAGGGAGCTG	GGTGTCTGTTTGCCCACTTT	104	100%	0.998
SOD	Superoxide Dismutase	JF825518	GGAGATCTTGGCAATGTTCAG	CAGTAGCCTTGCTCAGTTCATG	166	97%	0.991
CAS	Cellular Apoptosis Susceptibility Protein	JF825520	CTACAACCACTACCTGTTCGAGT	CAGGGACATGATCTGGAACAC	169	100%	0.995
CARP	Cell Cycle and Apoptosis Regulatory 1 Protein	JF825519	GCCAAGAGTGGGAAGTTTGAC	GAACATTTCATTGAACAATTCTGC	126	98%	0.997
IAP	Inibitor of Apoptosis Protein	JF825521	CAGGATTCTTCTACACAGGCAG	CCATTTCTTGTGTTCTCCCC	108	100%	0.988
HSP70	Heat Shock Protein 70	JF825515	CTTCGTTTGGTATCCATGTTGGTA	CTCTGTGTCCTGGTAGGCGAC	130	100%	0.997
HSP40	Heat Shock Protein 40	JF825514	GGATTATTATAAAGTGCTGGGG	GTCACTAAGTACATCATAGGCCTC	163	100%	0.996

Table 1 shows Pubmed accession numbers, primer sequences, amplicon sizes (base pair), oligo efficiencies (E) and correlation factors (R^2^) of the reference gene and genes of interest.

### Reverse Transcription-Quantitative Real Time Polymerase Chain Reaction (RT-qPCR)

RT-qPCR experiments were performed in a Chromo4 TM Real-time Detector (Biorad) thermal cycler, whereas fluorescence was measured using the Opticon Monitor 3.1 (Biorad). PCR volume for each sample was 25 µl, with 1× of Fast Start SYBR Green Master Mix (Roche), 2 µl of cDNA template and 0.7 pmol/µl for each oligo. The RT-qPCR thermal profile was obtained using the following procedure: 95°C for 10 min, 40 times 95°C for 15 sec and 60°C for 1 min, 72°C for 5 min. The program was set to reveal the melting curve of each amplicon from 60°C to 95°C, and read every 0.5°C. All RT-qPCR reactions were carried out in triplicate to capture intra-assay variability. Each assay included three no-template controls (NTC) for each primer pair. Five serial dilutions of cDNA were used to determine reaction efficiencies for all primer pairs. These efficiencies (Table 1) were calculated generating for each oligonucleotide pair standard curves with at least five dilution points by using the Cycle Threshold (Ct) value versus the logarithm of each dilution factor and using the equation E = 10^−1/slope^. As for previous studies [18], a 1∶100 template dilution (4±2 ng) was used for RT-qPCR experiments, in order to allow almost all gene amplifications to fit in the optimal detection window (from 15 to 25 cycles). All analyzed data were covered by the window defined by the standard curve generated for the calculation of the efficiency for each oligo pair.

Expression levels of each target gene in the tested experimental conditions (animals fed on *S. marinoi* and *C. socialis*) were compared to the control condition (animals fed on *R. baltica*) using the REST tool (Relative expression software tool) [49]. Data were normalized using the ribosomal protein S20, which had previously been identified as the best reference gene under different experimental conditions [18]. In the present analysis, the ribosomal protein S20 was confirmed to be stable, showing a variability always lower than ±1 cycle. The 1 x-fold expression level was therefore chosen as the threshold for significance of target genes. However, to validate our results, a statistical analysis was also performed using GraphPad Prism version 4.00 for Windows (GraphPad Software, San Diego California USA).

## Results

**Figure 2:**
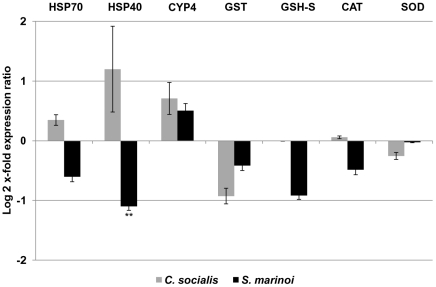
Expression levels of genes involved in stress and defense systems in the copepod *Calanus helgolandicus*. Changes in expression levels of Heat shock protein 70 (HSP70) and 40 (HSP40), Cytochrome P450-4 (CYP4), Glutathione S-Transferase (GST), Glutathione Synthase (GSH-S), Catalase (CAT) and Superoxide Dismutase (SOD) genes in *C. helgolandicus* fed either unialgal diets of *Skeletonema marinoi* (*S. marinoi*) or *Chaetoceros socialis* (*C. socialis*) compared to expression levels in females fed on the control *Rhodomonas baltica* (represented in the figure by x-axis) (** with p value<0.01, students't-test, GraphPad Software). The ribosomal protein S20 was used as reference gene to normalize the data.

**Figure 3:**
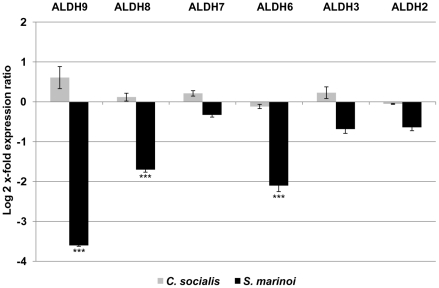
Relative gene expression levels of aldehyde dehydrogenases (ALDH) in the copepod ***Calanus helgolandicu*** **s.** Changes in ALDH2, ALDH3, ALDH6, ALDH7, ALDH8 and ALDH9 gene expression levels in *C. helgolandicus* females fed either unialgal diets of *Skeletonema marinoi* (*S. marinoi*) or *Chaetoceros socialis* (*C. socialis*) compared to expression levels in females fed on the control *Rhodomonas baltica* (represented in the figure by x-axis) (*** with p value<0.001, students't-test, GraphPad Software). The ribosomal protein S20 was used as reference gene to normalize the data.

**Figure 4:**
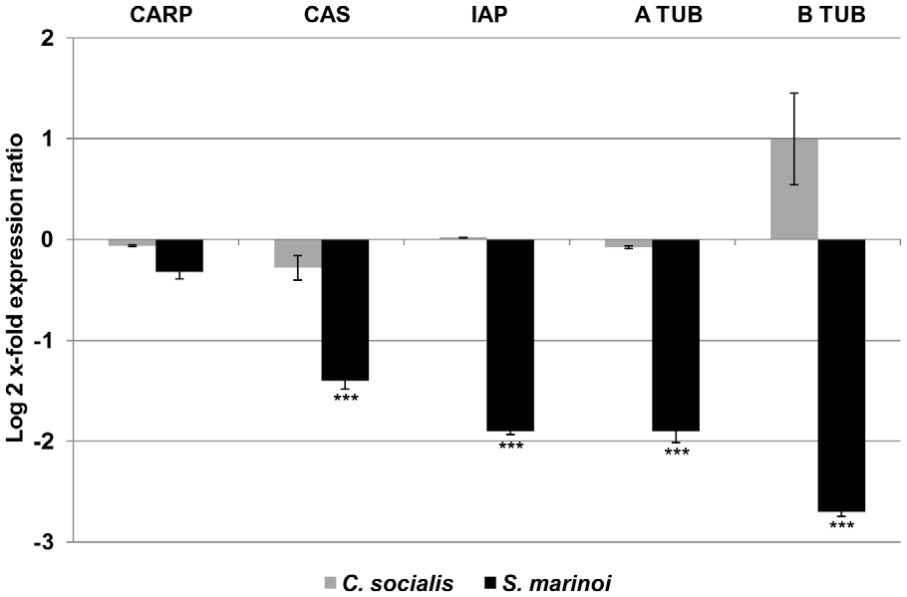
Expression analysis of genes involved in apoptosis and mitotic spindle formation in *C. helgolandicus*. Changes in expression levels of Cell Cycle and Apoptosis Regulatory 1 Protein (CARP), Cellular Apoptosis Susceptibility Protein (CAS), Inhibitor of Apoptosis Protein (IAP), and Alpha and Beta tubulins (ATUB and BTUB) genes in *C. helgolandicus* fed either unialgal diets of *Skeletonema marinoi* (*S. marinoi*) or *Chaetoceros socialis* (*C. socialis*) compared to expression levels in females fed on the control *Rhodomonas baltica* (represented in the figure by x-axis) (*** with p value<0.001, students't-test, GraphPad Software). The ribosomal protein S20 was used as reference gene to normalize the data.

The effects of diatom diets on *C. helgolandicus* females were evaluated by analyzing expression levels of genes involved in generic stress responses, defense systems, aldehyde detoxification or apoptosis regulation in other organisms (Figure 1). *C. helgolandicus* females fed the strong oxylipin and PUAs-producing diatom *S. marinoi* showed a general pattern of reduction in the expression levels of almost all the selected genes compared to females fed the control flagellate *R. baltica* (Figure 2, 3, 4). Both HSP40 and HSP70 transcript levels were reduced even if the change was only significant for HSP40 (p value<0.01, students't-test, GraphPad Software) (Figure 2). Expression levels of these genes in animals fed *C. socialis* did not change significantly. Enzymes involved in phase I and phase II reactions and anti-oxidant activity (CYP4, GST, GSH-S, CAT and SOD) did not show any significant changes in their expression levels in animals fed both diatom diets.

On the contrary, all six ALDH isoforms (ALDH2, ALDH3B1, ALDH6, ALDH7, ALDH8, and ALDH9) had lower expression levels in *C. helgolandicus* fed *S. marinoi* than those fed the control diet (Figure 3). However, only ALDH6, ALDH8 and ALDH9 were significantly affected, showing a 2–3 fold reduction in their expression levels (p value<0.001, students't-test, GraphPad Software). On the contrary, the expression levels for the same genes in copepods fed *C. socialis* did not show significant changes, indicating that these genes were not affected by this diatom diet.

Of the three proteins involved in apoptosis regulation (CARP, CAS and IAP) CAS and IAP expression levels were strongly reduced by *S. marinoi* compared to the control *R. baltica* and to *C. socialis*. In particular, CAS and IAP showed a significant 2-fold reduction (p value<0.001, students't-test, GraphPad Software) (Figure 4), while changes in CARP expression levels were close to zero. Gene expression profiles of alpha and beta tubulins, essential proteins for mitotic spindle formation, did not vary significantly in copepods fed on *C. socialis* compared to the control *R. baltica*. In contrast, *C. helgolandicus* fed *S. marinoi* showed a significant reduction of about 2-fold for alpha tubulin and 3-fold for beta tubulin (p value<0.001, students't-test, GraphPad Software) (Figure 4).

## Discussion

Our results show that two days (2 d) of feeding of *C. helgolandicus* on *S. marinoi* is sufficient to inhibit a series of genes involved in generic stress response, aldehyde detoxification and apoptosis regulation. Of the analyzed transcripts at least 50% were strongly reduced (ALDH9, ALDH8 and ALDH6, CAS, IAP, HSP40, alpha- and beta-tubulin) with a *S. marinoi* diet, while no significant gene expression changes were observed in animals fed on the other diatom *C. socialis*. Previous studies have shown that after 2 d of feeding on *S. marinoi* egg viability in *C. helgolandicus* is still high (>90%) and decreases to about 50% after 3 d [3]. On the contrary, with a *C. socialis* diet, egg viability is high (90%) even after 3 d [3] indicating that this diatom is less toxic for copepod reproduction. Fontana and co-workers [3] concluded that the lower toxicity of *C. socialis* was due to the fact that this diatom does not produce PUAs but only low quantities of hydroxyl-acids and epoxy-alcohols compared to *S. marinoi*. Our results indicate that there is a significantly different response in gene expression patterns in *C. helgolandicus* fed on these two diets thereby offering a possible explanation as to why in nature certain diatom blooms may be more toxic for copepods [1], [16] compared to others [50], [51].

Until now, gene expression studies in copepods have been performed after exposure to various toxicants such as naphtalene [52], diethanolamine [53] and mono ethanol amine (MEA), water-soluble fractions of oil (WSFs), trace metals [54] and endocrine-disrupting chemicals [55] (as reviewed by [56]). In most of these studies [52], [53], [57], [58], detoxification gene expression levels increased when copepods were challenged with toxicants, but in our case there was a general pattern of decrease and both general stress systems and specific responses seemed to be inhibited.

Both HSP70 and HSP40 expression levels decreased in females fed on *S. marinoi* suggesting a reduction in chaperone activity in the folding of new proteins, repairing of unfolded and damaged proteins, and inhibition of protein aggregations, thereby leading to an increase in cellular damage. Romano et al. [59] have recently shown that sea urchins activate HSP70 when challenged with low concentrations (0.25 µg/ml) of the PUA decadienal thereby protecting embryos against the toxic effects of this aldehyde. This up-regulation was only found at 9 h post fertilization (hpf), while at 5, 24 and 48 hpf, expression levels were comparable to the control. Small changes in HSP70 mRNA levels were found in *C. finmarchicus* after naphtalene exposure [52], while Rhee and co-workers [60] showed a concentration-dependent increase in the expression of HSP70 transcripts after exposure to trace metals (i.e. copper, silver, and zinc), with an increase caused by bisphenol A (BPA) and a decrease by 4-nonylphenol (NP) and 4-t-octylpheno (OP).

Enzymes involved in antioxidant cell activity (GST and GSH-S) and in free radical detoxification (CAT and SOD) did not show significant expression level changes in *C. helgolandicus* fed either of the two diatom diets indicating that they were not involved in the defense response of this copepod species, at least after two days of exposure. Kozlowsky-Suzuki and co-workers (2008) also suggested that GST enzymatic activity did not seem to play a role in detoxification of copepods exposed to toxic dinoflagellate algae: *Alexandrium minutum* and *Alexandrium tamarense*, which contained Paralytic Shellfish Poisoning (PSP) toxins, and the dinoflagellate *Prorocentrum lima* with Diarrhetic Shellfish Poisoning (DSP) toxins. On the contrary, GST expression levels were affected in the copepod *Calanus finmarchicus* after exposure to naphthalene [52] and diethanolamine (DEA) [53] and in the copepod *Tigriopus japonicus* exposed to trace metals and hydrogen peroxide (H_2_O_2_) [58]. The responses were mainly concentration- and time-dependent and varied with the tested stressors. Hansen and co-workers [52] showed that only the lowest naphthalene concentration in *C. finmarchicus* led to increased mRNA levels of the ROS detoxification enzymes SOD and CAT, but no effects were found at medium and high concentrations, indicating no clear evidence for general cellular oxidative stress following naphthalene exposure. On the other hand, the transcription levels of the antioxidant glutathione synthase (GSH-S) and Cu/Zn-superoxide dismutase (SOD) changed with a concentration-dependent pattern following exposure to DEA in the same copepod species [53]. SODs expression levels in the harpacticoid copepod *Tigriopus japonicus* increased only at the highest heavy metal concentrations tested and showed different responses to endocrine disruptor chemicals (EDCs) depending on the specific stressor and its concentration [61].

The aldehyde dehydrogenase family, which should detoxify and inactivate aldehydes, was almost switched off in animals fed on the *S. marinoi* diet. Gene expression levels were significant reduced by about 4-fold for ALDH9 and 2-fold for ALDH8 and ALDH6. Until now, ALDH gene expression levels have been mostly analyzed in humans and this is the first time that they have been analyzed in a copepod species. It is widely known that ALDH are involved in protecting cells from the deleterious effects of xenobiotics and endogenous aldehydes such as those derived from lipid peroxidation [62]. Our results suggest that this enzyme family in copepods fed on the diatom *S. marinoi* is probably not able to detoxify high levels of toxic diatom aldehydes, and that therefore there is accumulation of these compounds in body tissues or formation of adducts.

Selected apoptosis regulatory proteins were also affected by the *S. marinoi* diet. The two proteins whose function in humans is to inhibit apoptosis, CAS and IAP, were significantly down-regulated by about 2-fold, yet apoptotic processes were not inhibited, at least after 2 d of feeding. The fact that CARP, a protein generally associated with an increase in apoptosis [63], did not respond to the diet suggests that there was no clear apoptosis induction in adult females in our experimental conditions. Buttino *et al.* [64] using aldehyde-encapsulating liposomes observed apoptotic regions in copepod female gonads only after 9 d of feeding. We therefore assume that induction of pro-apoptotic proteins may only occur after longer exposure to the toxic diet.

Alpha and beta tubulins, structural subunits of MTs and the targets of many natural toxins, were previously reported to be 2-fold and 3-fold down-regulated, respectively, with a *S. marinoi* diet [18]. Here we confirm our previous findings and also show that *C. socialis* does not induce the same pronounced reduction in the expression levels of these two genes. Future studies on PUAs-tubulin interactions may clarify if alpha and beta tubulins are the targets of toxic *S. marinoi* metabolites or if their gene expression reduction is a secondary effect of PUAs toxicity.

Our results provide molecular evidence for the toxic effects of certain diatom diets on grazers, showing that primary defense systems that should be activated to protect copepods against dangerous algae are inhibited. This exploratory study is currently being extended with the creation of a suppression subtractive hybridization library for *Calanus helgolandicus* which may further help to clarify which genes are differentially expressed in response to the ingestion of some diatom species. Given the importance of diatom blooms in nutrient-rich aquatic environments these preliminary results offer a plausible explanation for the inefficient use of a potentially valuable food resource—the spring diatom bloom—by some zooplankton [1], [16]. Also terrestrial plants produce toxins which cause abortions, reproductive dysfunction and occasional birth defects when ingested by certain grazers [65] suggesting that interactions among organisms are regulated by similar mechanisms in terrestrial and marine ecosystems.
